# Precedent Autonomy and Surrogate Decisionmaking After Severe Brain Injury

**DOI:** 10.1017/S0963180120000286

**Published:** 2020-10

**Authors:** MACKENZIE GRAHAM

**Keywords:** disorders of consciousness, brain injury, surrogate decisionmaking, advance directive, substituted judgment

## Abstract

Patients with disorders of consciousness after severe brain injury need surrogate decision makers to guide treatment decisions on their behalf. Formal guidelines for surrogate decisionmaking generally instruct decision makers to first appeal to a patient’s written advance directive, followed by making a substituted judgment of what the patient would have chosen, and lastly, to make decisions according to what seems to be in the patient’s best medical interests. Substituted judgment is preferable because it is taken to preserve patient autonomy, by using a patient’s past wishes and values to reconstruct what they would have chosen for themselves. In this paper, the author argues that for a certain population of patients, the standard interpretation of substituted judgment cannot ensure the preservation of patient autonomy. Patients with “covert awareness” may continue to have values and an authentic sense of self, which may differ from their past values and wishes. Accordingly, surrogate decision makers should make decisions based on how the patient is likely to experience their condition in the present, rather than their past wishes and values.

## Introduction

Frieda, a 32-year-old female, suffers a severe traumatic brain injury when she is struck by a motor vehicle while riding her bicycle. She is rushed to the hospital, and her condition is stabilized by emergency physicians, but she remains in a coma for 5 days. Her husband, acting as her surrogate decision maker, is informed by her neurologist that her prognosis is uncertain, and he elects to continue life-sustaining treatment.

After 2 months, she has emerged from her coma and is diagnosed as having unresponsive wakefulness syndrome (UWS)—also referred to as the vegetative state (VS)—showing no evidence of awareness of herself or her environment during bedside examination. However, an electroencephalogram has detected a P300 signal in response to a recording of her name, which has been found to be predictive of recovery in posttraumatic VS.^1^

At this stage after her injury, it remains unclear to what extent Frieda might recover. Her husband believes that she would not want to continue living in a severely disabled state, given the feelings she had expressed about the prolonged period of disability her grandmother experienced prior to her recent death. Her parents and sister believe that there is a reasonable chance of meaningful recovery, and that with the right support, she could have an acceptable quality of life even in a severely disabled state. However, none of Frieda’s family is really sure how to proceed.

## Disorders of Consciousness

Few cases exemplify the challenge of surrogate decisionmaking more than patients with prolonged disorders of consciousness. Disorders of consciousness are a collection of transitory syndromes, encompassing coma, UWS, and the minimally conscious state, and are considered “prolonged” after more than 4 weeks postinjury.^2^ Diagnosis of a particular syndrome is based on the presence or absence of qualifying signs of wakefulness and awareness. A patient in a coma is neither awake nor aware, whereas a patient with UWS remains awake, but unaware of themselves or their surroundings. Patients in the minimally conscious state are awake and demonstrate intermittent and fluctuating levels of awareness. Although they share certain essential features, patients within each syndrome may be very different. Diagnosis is typically made from a bedside behavioral assessment using a diagnostic tool like the Coma Recovery Scale-revised, and rates of misdiagnosis remain high.^3^

Several landmark studies over the past decade have caused a dramatic shift in our understanding of disorders of consciousness. In the most widely cited case, researchers demonstrated that a patient who had been diagnosed as being in a VS could understand spoken commands, and respond to them, by modulating her brain activity.^4^ After being placed in a magnetic resonance imaging scanner (MRI), the patient was instructed to imagine playing tennis, or imagine navigating their home. During each imagery period, functional MRI detected activity in specific brain areas—the same as that observed in healthy subjects—which would then cease when the patient was instructed to stop imagining. These results provided compelling evidence that the patient was, despite all outward appearances, still conscious.

Subsequent studies have determined that as many as 15% of patients rigorously diagnosed with UWS are covertly conscious.^5^ ^,^^6^ ^,^^7^ ^,^^8^ Patients fitting this profile—behaviorally nonresponsive but capable of command-following using functional neuroimaging—are referred to as having “cognitive motor dissociation,” and encompass a wide range of potential cognitive capacity.^9^ Command-following studies provide strong evidence of a preserved capacity for language comprehension, attention, response selection, and working memory, whereas other neuroimaging studies in these patients have demonstrated preserved executive function, and “theory of mind.”^10^ ^,^^11^ A subset of patients have even used functional neuroimaging to correctly communicate answers to “yes or no” questions.^12^

Despite their preserved consciousness, patients with cognitive motor dissociation are presumed to lack decisionmaking capacity. Although it is possible that such a patient could retain the necessary understanding, appreciation, and reasoning to make a particular decision, and, in the case of patients who can communicate using functional neuroimaging, the means to convey this decision to others, the extent of their injuries makes this highly uncertain. Additionally, the limited ways in which these patients can communicate (if at all) makes assessing decisionmaking capacity challenging. For these reasons, decisions are made on their behalf by surrogate decisionmakers.

To date, the vast majority of patients who have demonstrated covert awareness have been several months, or even years, postinjury when awareness was verified using functional neuroimaging. These patients are generally physiologically stable, and living in a long-term care home or with family members. Further recovery of function is unlikely (though not impossible). At this stage, end-of-life decisions tend not to be at the forefront for surrogate decisionmakers. Decisions about whether to pursue life-sustaining treatment, such as tracheostomy or the insertion of feeding tubes, are typically made relatively soon after injury, and it has been uncommon for families participating in this research to subsequently withdraw life-sustaining treatment from their family members. Accordingly, the discovery of covert awareness in patients who are chronically behaviorally nonresponsive may not significantly impact surrogate decisionmaking with respect to withdrawing treatment.

However, the potential for covert awareness in as many as 15% of behaviorally nonresponsive patients may complicate surrogate decisionmaking in the acute stage, when treatment decisions are more pressing. Because prognosis after severe brain injury is highly uncertain, surrogate decisionmakers are faced with a difficult task. In the hours or days immediately after injury, when the patient remains physiologically unstable, surrogates must decide to pursue treatment or opt instead for palliative care, without knowing whether or not the patient will make a good recovery (i.e., a quality of life the patient would find acceptable). If the patient survives their injuries and becomes physiological stable, recovery of function remains uncertain. The likelihood of significant functional improvement after severe brain injury diminishes over time; recovery of consciousness is considered unlikely 12 months postinjury for patients suffering a traumatic brain injury, and 3 months postinjury for those suffering an anoxic brain injury, although isolated cases of recovery of consciousness several years after injury have been documented .^13^ During this period, surrogate decisionmakers will need to decide how the patient’s treatment should be managed, including in some cases whether to withdraw life-sustaining treatment, or withhold a potentially life-prolonging intervention.

At present, functional neuroimaging to detect covert awareness occurs in only a handful of research institutions globally, making it inaccessible to the majority of behaviorally nonresponsive patients. However, in the 2018 update to its practice guidelines for patients with disorders of consciousness, the American Academy of Neurology recommends that when there is continued ambiguity regarding evidence of conscious awareness, or where confounders to a valid clinical diagnostic assessment are identified, clinicians should use multimodal evaluations to detect conscious awareness, including functional neuroimaging.^14^ This is an important step toward making functional neuroimaging to detect covert awareness more widely available.

These guidelines also point out that functional neuroimaging studies return negative findings in the majority of patients diagnosed as having UWS through behavioral assessment. Although a negative result on a functional neuroimaging test does not entail that a patient lacks awareness (only that no evidence of awareness was detected), the available evidence suggests that functional neuroimaging will not result in a change in diagnosis for most patients. More sensitive methods of detecting covert awareness are needed, and continue to be developed by researchers. For the purposes of this discussion, however, I will focus on patients who have demonstrated evidence of covert awareness via functional neuroimaging or other modality, or for whom such an assessment is indicated (because there is continued ambiguity regarding evidence of conscious awareness, based on serial neurobehavioral assessment).

Thus, in addition to navigating the uncertain trajectory of patient recovery, surrogate decisionmakers must also account for the fact that in a minority of cases, the patient may be covertly aware. In the absence of awareness, patients cannot experience pain or pleasure—or more broadly—suffering or enjoyment. Conversely, the presence of covert awareness means that patients have the capacity to experience the world in some way. Yet we know very little about what this experience is like. This has led some to worry that patients might be suffering tremendously in this state; conscious of the world around them, but unable to move or speak.^15^ ^,^^16^ How should surrogate decisionmakers navigate the difficult task they have, in light of these worries?

## The Standard View of Surrogate Decisionmaking

The standard view of surrogate decisionmaking adopted in most legal statutes uses a hierarchical framework, beginning with an appeal to a patient’s written advance directive.^17^ ^,^^18^ In cases where a formerly competent patient expressed a preference for or against certain interventions through an advance directive, the role of the surrogate is to ensure that these preferences are adhered to as much as possible. When no advance directive exists, the task of the surrogate is to make a “substituted judgment” on behalf of the patient, by reconstructing what the patient would have decided in the circumstances, if they were competent. If a substitute judgment is not possible—either because the patient never expressed a preference relevant to the decision at hand, or because the patient is not known to the surrogate decisionmaker—the surrogate should act according to what they believe to be in the patient’s best interests. This “best interests standard” appeals to a general conception of interests (e.g., minimizing suffering and restoration of physical capacities), drawing from what a reasonable person would be thought to want in the circumstances.

In practice, surrogate decisionmaking often departs from the standard model. When making decisions on behalf of patients with disorders of consciousness, surrogate decisionmaking may reflect a combination of different considerations.^19^ ^,^^20^ ^,^^21^ ^,^^22^ Surrogates may use conversations with physicians about the patient’s prognosis, or the possible trajectory of their recovery or decline, to make decisions. Surrogates also tend to weigh the burdens of continued treatment on the patient. Is the patient suffering physically, emotionally, or mentally? The patient’s anticipated quality of life is also an important consideration, as well as the patient’s prior expressed wishes, whether they “would have wanted to live like this.” Surrogates may draw on their beliefs about the patient’s personality (“she is a fighter”), or religious beliefs. Surrogates may also consider the expected burden of caring for the patient, or what the surrogates themselves would want, if they were the patient.

Surrogate decisionmaking becomes challenging when these factors do not lead to a clear conclusion, as is often the case in this context, and as we see in Frieda’s case. Her uncertain prognosis means it is unclear if she will recover, and to what extent. The possibility that she is covertly aware means that she could be experiencing her condition, though it is unclear if she is suffering. And if she is covertly aware, we might be unsure whether the fact that she previously expressed not wanting to live in such a state is a sufficient reason to withdraw her from treatment.

Adhering to the standard view of surrogate decisionmaking provides one way of resolving this conflict. The surrogate should make a substituted judgment, based on Frieda’s prior expressed preferences. Frieda had said in the past that she would not want to continue living in a severely disabled state, and her family should respect this decision and withdraw her from continued treatment.

In what follows, I will argue that appealing to the standard view in the case of behaviorally nonresponsive patients like Frieda is mistaken. This is not because I think that behaviorally nonresponsive patients should not be withdrawn from treatment. Rather, it is because appealing to the substituted judgment standard fails to account for a crucially important aspect of patients with covert awareness, which significantly weakens the underlying justification for its use. Substituted judgment presumes that reconstructing the decision a now-incompetent patient would have made, based on an advance directive, or their prior decisions and values, is the best way to respect their autonomy. However, as I will argue, in the case of behaviorally nonresponsive patients with covert awareness, we have reason to question this presumption. If my argument is successful, surrogate decisionmakers should appeal to a different decisionmaking standard when acting on behalf of behaviorally nonresponsive patients.

## Foundations of the Standard View of Surrogate Decisionmaking

Most Western countries, particularly the United States, place a high value on the autonomy of patients to make their own decisions about medical treatment. Because the burden of any treatment decision is primarily borne by the patient, patients have the right to choose which treatments they will or will not accept. Surrogate decisionmaking proceeds from this starting point: when a patient cannot be relied upon to decide for themselves about their treatment and care, how can we protect their general right to make their own decisions? A natural strategy is to look at the wishes the patient expressed while still competent. If they cannot decide for themselves now, the best alternative is to appeal to the choices they made in the past. Accordingly, surrogate decisionmaking typically begins by looking to a patient’s written advanced directive—a statement indicating the treatments a patient would and would not accept in various circumstances.

A highly influential argument for the primacy of advanced directives for surrogate decisionmaking comes from Ronald Dworkin.^23^ On his view, a person’s well-being—how well their lives go, for them—depends on the satisfaction of “experiential” interests and “critical” interests. Experiential interests are those that we value because we like the experience of doing them; they are things that we find exciting, enjoyable, or pleasurable. Critical interests, by contrast, are concerned with those things we believe to be genuinely important for a good life. Rather than preferences for certain experiences, critical interests are judgments about what is valuable, about what people should want from their lives and without which they would be much worse off. It is in the pursuit of our critical interests that we make important decisions, and structure the narrative shape of our lives. Unlike experiential interests, critical interests are not temporally indexed. If I believe a certain kind of achievement will make my life better, for example, I will be indifferent about whether it has occurred in the past, or will occur in the future. Similarly, satisfying a critical interest can contribute to the value of my life, even if I am not aware of it.

Dworkin also suggests that although we want our lives to contain the right kind of experiences and achievements, we also want these experiences to have a kind of integrity. For Dworkin, a good life forms a coherent narrative structure, where the critical interests we value and pursue express our commitment to a certain kind of character. This commitment to a coherent life-narrative explains why people often care quite deeply about the circumstances in which they approach the end of life. People want their deaths to be in character with their unique life-narrative, to be consistent with the sense of self they have curated over the course of their lives. Accordingly, Dworkin argues, considerations of beneficence and respect for autonomy require that people be able to determine the ends of their lives, where possible.

How would Dworkin respond to a behaviorally nonresponsive patient like Frieda? Dworkin points out that it is generally accepted that competent individuals have the right to autonomy, and we should respect their decisions even when they appear not to be in the person’s best interests. We do this because we recognize the importance of autonomy to preserving the integrity of our character. We value autonomy because it protects our ability to lead a life structured by our own values and commitments. Even though people sometimes have inconsistent values, or make irrational decisions, it is important that we allow them to make decisions according to their sense of self.

When a person loses the capacity to choose according to a coherent sense of self, they have lost the capacity that autonomy is meant to protect, and their decisions no longer have authority which must be respected by others. However, we might still have a reason to respect those decisions they have made in the past, when they still had this capacity—what Dworkin calls “precedent autonomy.”^24^ By respecting a person’s precedent autonomy we continue to allow them to shape their life-narrative according to their own values and commitments, to live their lives in the way that they wanted to.

On Dworkin’s view of precedent autonomy, the reason we respect a patient’s past decision is not because we take this as good evidence of their interests in the present, but because we take it to be an expression of their autonomy. Thus, even if honoring an incompetent patient’s past decisions appears to conflict with her best interests, we ought to defer to the past decision because no subsequent expression of the patient’s autonomy has been made to replace it.

Dworkin also argues that we have reasons of beneficence to defer to a patient’s prior expressed wishes when making decisions on their behalf. He argues that when a person is entrusted to the care of another, that person has a right that decisions be made in their best interests. At first glance, this appears to conflict with the patient’s right to precedent autonomy; the carer’s judgment of the patient’s best interests might be at odds with the patient’s prior expressed wishes. It is here that Dworkin’s distinction between experiential and critical interests is important. Although a patient lacking decisionmaking capacity will continue to have experiential interests, they no longer have a clear sense of their critical interests. They do not have a sense of their life as a whole, or of the kinds of plans and goals which structure a life-narrative. Yet they continue to have critical interests, interests which are a continuation of those critical interests they had while competent. He argues that when we consider the entire character of their lives, including those critical interests the patient had while competent, we see that we must judge the patient’s critical interests as they did when they were competent. Because our critical interests are morally weightier with respect to our overall well-being than our experiential interests, respecting a patient’s past critical interests best promotes their well-being overall.

## Advance Directives

Dworkin argues that the most immediate way to respect critical interests is to honor a patient’s written advance directives. In practice, however, advance directives often fall short of this ideal. First, most people simply do not complete them. Although research suggests that many people know about them, only about 25%–30% of people have them, even when educational interventions have been designed to facilitate their use.^25^ ^,^^26^ Written advance directives are also more common in elderly people, although patients who regain covert awareness after severe brain injury are often younger or middle-aged adults, making it likely that the patients under discussion will not have completed an advance directive.

Not all advance directives contain meaningful or useful information. Most advance directives are broad, attempting to cover a wide range of possible treatment scenarios. Accordingly, they usually provide only general instructions for how the patient would like to be treated (e.g., “I would not want to receive life-sustaining treatment”), but lack the level of detail which would be helpful to surrogate decisionmakers in real treatment situations. Most patients are unlikely to have given serious thought to the possibility of behavioral nonresponsiveness with the possibility of covert awareness after a severe brain injury, meaning the majority of advance directives likely will not provide specific instructions for these circumstances.

Even a detailed advance directive is not useful if patients do not understand the medical decision they are making. When competent patients make medical decisions in the present, we protect their interests by ensuring that they have an adequate understanding of the information relevant to their treatment decision; they should have at least a basic understanding of the nature of the condition, as well as potential benefits and harms of particular treatments. Yet research suggests that the decisions articulated in advance directives are often uninformed.^27^ ^,^^28^ Directives may be internally inconsistent, and patients may exhibit a lack of understanding about how specific interventions cohere with their broader treatment preferences. We do not respect a patient’s autonomy or life-narrative by binding them to uninformed decisions they made in the past.

In the case of covert awareness, meeting this threshold of understanding can be difficult. Because we currently know very little about how these patients experience the world, assumptions healthy people make about the condition may be uninformed. A person creating an advance directive should understand that these patients are incapable of voluntary behavior, and completely dependant on others for their care. But they should also be aware that this condition is not necessarily physically painful, and that patients may be capable of experiencing pleasure and various other forms of enjoyment, depending on their cognitive capacities. The level of care and support these patients receive is also likely to be a significant determining factor in their overall quality of life.

Preferences for life-sustaining treatment may also change over time, without people necessarily being aware of this change. People generally do a poor job of predicting their preferences and emotional reactions to future events, particularly when they are unfamiliar, which is likely to be the case for most situations in which an advance directive would be invoked.^29^ ^,^^30^ Research suggests that rather than resulting from reflective deliberation, most decisions about treatment are made on the spot by the decision maker, and can be heavily influenced by biases, framing effects, and other contextual factors.^31^ Again, this suggests that rather than being an expression of a person’s autonomous values and commitments about how their lives should go, a written advance directive is often a haphazard recording of a person’s potentially unreflective preferences at a particular time.

## Substituted Judgment Standard

The practical problems with advance directives often require recourse to the substituted judgment standard. Like an advance directive, the appeal of substituted judgment is that it ostensibly supports the patient’s autonomy, even after decisionmaking capacity has been lost. By making decisions on the patient’s behalf according to what they would have chosen for themselves, we continue to promote their autonomously chosen critical interests. Again, the general acceptance of the substituted judgment standard amongst clinicians and many bioethicists reflects the fundamental importance of self-determination in Western medical decisionmaking.

Despite its widespread use, the substituted judgment standard presents many of the same concerns as advance directives. Substituted judgment proceeds from the assumption that an individual’s wishes and preferences are at least somewhat stable, such that an expressed preference will still be applicable at some future time. Yet individuals’ preferences regarding life-sustaining treatment often change over time, with research indicating that people are more likely to change their minds about treatment decisions they have not articulated in an advance directive.^32^ ^,^^33^ ^,^^34^ This suggests that those people who are most likely to require substituted judgment are those for whom past preferences are less likely to be applicable in the present.

Introducing another party to infer or speculate about what the patient would have decided also introduces new sources of potential error. Surrogates may have difficulty identifying where their own needs and values differ from those of the patient, or they may be influenced by decisionmaking biases (e.g., the “status quo bias,” where decisionmakers prefer the status quo to making changes to treatment), resulting in under or overtreatment.^35^ ^,^^36^ The stress, anxiety, and uncertainty associated with surrogate decisionmaking has also been speculated to reduce surrogates’ ability to make accurate judgments.^37^ As a result, surrogate decisionmakers are often inaccurate in representing patients’ medical preferences. When comparing what participants chose for themselves in hypothetical situations, and what surrogate decisionmakers predicted they would choose, surrogates are correct approximately 68% of the time.^38^

Although these problems present a difficult challenge to overcome, I think patients with covert awareness present a deeper issue for substituted judgment. Recall that on Dworkin’s view, the right to precedent autonomy is grounded in the value of a person’s integrity; their capacity to lead their lives “out of a distinctive sense of their own character.”^39^ In paradigm cases of decisionmaking incapacity, such as advanced Alzheimer’s, the loss of decisionmaking capacity is taken to be roughly contemporaneous with the loss of the ability to conceive of one’s critical interests. Although they may have periods of greater or lesser lucidity, dementia brought on by Alzheimer’s is a progressive decline in cognitive capacity, until patients no longer have a clear and coherent sense of themselves. At this point, they can no longer “act out of genuine preference or character or conviction,”^40^ and as a result, no longer have the right to autonomy. The underlying assumption is that the capacity to make autonomous decisions—and the right to have them respected—is tightly bound with the capacity to have genuine preferences about the shape of one’s life (i.e., to have critical interests).

But behaviorally nonresponsive patients are not like patients with advanced Alzheimer’s. These patients do not undergo a progressive decline in their cognitive capacities, but rather, have experienced a shearing of their cognitive capacities, followed by an unknown level of recovery. In the Alzheimer’s case, we need to refer to a patient’s past expression of their critical interests, in order to preserve and maintain their life-narrative. But it is possible that a behaviorally nonresponsive patient with covert awareness might continue to have genuine preferences about the shape of their lives, even without having decisionmaking capacity. Put another way, covertly aware patients lack agency—they lack the means to purposefully act on their environment—but they may still have the capacity to formulate judgments or decisions which express genuine values and commitments. If so, a patient’s past expression of her critical interests would not be the best guide to decisionmaking on her behalf. In fact, such a patient might have critical interests in the present which are different than the critical interests they expressed in the past, and they might no longer endorse those past critical interests.^41^

This possibility presents a difficulty not only for Dworkin’s view, but similar views of surrogate decisionmaking which ground substituted judgment in the patient’s prior values and life narrative. For example, John Philips and David Wendler propose “the endorsed life” interpretation of substituted judgment, according to which surrogate decisionmaking ought to promote the life that the patient valued for themselves.^42^ On this view, we respect the autonomy of a now-incompetent patient by helping them to bring about the life they wanted to lead. Of course, the assumption is that the values which guided the patient’s autonomous decisionmaking in the past have not been replaced by different or contrary values in their postinjury state. Although this may be a reasonable assumption in the case of advanced dementia, it may not be in the case of behaviorally nonresponsive patients with covert awareness.

Brudney and Lantos^43^ offer an alternative view, which emphasizes a distinction between agency and authenticity, as components of autonomy. Agency refers to a person’s capacity to choose on the basis of reasons, where each choice we make is an exercise of our agency. Authenticity, on the other hand, refers to “constructing one’s life in accordance with one’s distinctive beliefs and values,”^44^ and must be exercised over the course of many decisions. Brudney and Lantos argue that agency and authenticity may diverge in some cases, such as when a patient refuses treatment for reasons which seem inconsistent with the values they have lived by. Although respect for an individual’s agency is important, allowing the mere exercise of an individual’s choice does not seem more valuable than preserving their life. Thus, it is only when agency is consistent with authenticity that we ought to respect a patient’s decision to refuse life-sustaining treatment. If we extend this argument to cases of surrogate decisionmaking, the substituted judgment of surrogates would need to reflect the patient’s authentic self, and not merely a prior expression of her agency. Again, the problem presented by behaviorally nonresponsive patients with covert awareness is evident. If these patients continue to have distinctive beliefs and desires about how their lives should go, it would be mistaken to defer to their prior expressed decisions, or the values and character they expressed in the past, if these no longer reflect their authentic self.

## Evidence for Critical Interests in Patients with Covert Awareness

What reason do we have for thinking that patients with covert awareness continue to have the capacity to form new critical interests? As described above, the mental imagery task requires the exercise of a range of cognitive capacities: sustained attention (required to maintain focus through each task), language comprehension (required to understand the instructions of the researchers), response selection (required to switch between alternative task requirements), decisionmaking and execution skills (required to decide whether to comply with the task instructions and carry out the mental task), and working memory (required to remember task instructions, and which task to perform). As Davinia Fernandez-Espejo and Adrian M. Owen argue, “these are all aspects of ‘top-down’ cognitive control that are typically associated with normal levels of conscious awareness.”^45^

Those patients who have used mental imagery for yes/no communication demonstrate evidence of additional cognitive capacities. For example, one patient was able to correctly identify his name, suggesting self-identity, and correctly identify the year and his location, suggesting orientation in space and time. This patient also correctly identified the name of his personal support worker, whom he had only met after his accident, suggesting the capacity to form new memories. Finally, the ability to answer that he enjoyed watching hockey on TV, and that he was not in pain, demonstrates a capacity for personal preferences, and subjective experience.^46^

Other approaches to functional neuroimaging reveal further cognitive capacities. In an elegant series of experiments, Lorina Naci and colleagues compared the brain responses of healthy participants, and two patients diagnosed as being in a VS, as they were shown a suspenseful movie.^47^ They found that one of the VS patients demonstrated a highly similar brain response to the healthy controls, suggesting a similar experience of the movie (this patient was later found to have cognitive motor dissociation through an independent neuroimaging scan). Similar experiments using audio-only stimuli have also generated compelling evidence of a highly similar conscious experience between healthy controls, and some behaviorally nonresponsive patients.

Following the complex plot of a suspenseful film requires various cognitive capacities. In addition to the retention of visual and auditory function, patients must also retain executive function. Executive function is a process which allows us to integrate auditory and visual information, and our prior knowledge and experiences, into a meaningful whole. Understanding a complex narrative also requires a “theory of mind,” which allow us to infer others’ mental states and differentiate them from our own.^48^ Finally, the experience of suspense requires having certain beliefs about the past as well as certain beliefs or expectations about the future, and the capability to adjust these beliefs when new information is presented. The experience of suspense has been shown to recruit brain regions involved in making strategic inferences, and also involves future-directed cognitive processes.^49^ ^,^^50^ A behaviorally nonresponsive patient capable of experiencing a suspenseful stimulus could also be capable of organizing her own experiences according to a temporally coherent structure.

Taken together, this evidence warrants at least the possibility that patients with covert awareness continue to have a sense of their lives as a whole, and a distinctive sense of their own character. Thus, although they may not be capable of exercising their agency, they may continue to form and revise their critical interests in the present (this might be the case even if they remain cognitively impaired). If so, we can no longer be certain to be respecting their autonomy by honoring their advance directives, or making a substituted judgment based on the kind of life they valued, because those values may have changed. In other words, patients with covert awareness may retain the capacity to genuinely revise their life narrative. Similarly, appealing to a patient’s previously expressed critical interests is no longer clearly consistent with beneficence, if these critical interests are no longer endorsed by the patient in the present.

This possibility adds a serious complication to surrogate decisionmaking. Respect for a patient’s autonomy no longer clearly favors deferring to their prior decisions or values. Similarly, even if we are persuaded by Dworkin’s interpretation of beneficence, we might fail to decide in the patient’s best interests by appealing to their past critical interests.

At this point, one might reasonably ask the following: Suppose that some covertly aware patients do retain sufficient cognitive capacities that they could authentically revise their critical interests postinjury. Do we have reason to think that their current critical interests will be substantially different than their past critical interests? For example, if a patient expressed in an advance directive that they would not want to be kept alive in a state of complete dependence, or had lived the kind of life that strongly suggests they would not want to live in such a state, why should we not assume that this continues to be their critical interest?

Our critical interests reflect those things that we value in life, and it stands to reason that these would be relatively stable. It is also true, however, that our critical interests can be revised, often in light of transformative life experiences. Evidence from the quality of life literature supports the idea that our critical interests may undergo change after a serious illness or injury. “Response shift” refers to a recalibration, reconceptualization or reprioritization of a patient’s values and commitments in response to significant changes in their circumstances, in a way that affects their self-evaluation.^51^ ^,^^52^ ^,^^53^ A patient may change the value they assign to different elements of their life, or they may change their understanding of what a good life is, for them.

A particularly illustrative example of potential response shift is patients with locked-in syndrome. Locked-in syndrome is a condition in which patients are incapable of voluntary movement (except, in most cases, for vertical eye movement), or verbal communication, but remain completely cognitively intact. As we might expect, many healthy people would not want to be kept alive with locked-in syndrome. Jacob Gipson and colleagues found that 35.8% of healthy people would want treatment withdrawn if they were in this condition, although 38.9% were unsure. Conversely, studies have shown that the self-reported quality of life of locked-in patients is within the same general range as that of healthy individuals,^54^ ^,^^55^ with one study indicating that 72% of patients self-reported as happy.^56^

A few caveats are relevant here. First, as the authors of the study point out, the 72% figure may reflect a selection bias, as more well-adjusted patients might be more likely to participate in a quality of life-survey. Indeed, the same study found that 58% of patients would not want to be resuscitated in the event of cardiac arrest, although only 7% expressed a wish for euthanasia.^57^ Clearly, not all locked-in patients have a quality of life acceptable to them. Second, although both locked-in and covertly aware patients have similar motor limitations, locked-in patients are generally capable of some kind of communication. They have a means of expressing their preferences and engaging with others that is almost entirely unavailable to the majority of covertly aware patients. This difference alone is likely significant enough to prevent an “apples to apples” comparison between these populations.

Nevertheless, even if the self-reports of locked-in patients cannot be directly extrapolated to covertly aware patients, locked-in patients provide a compelling example of the potential of at least some patients with significant disabilities to adapt to their condition and maintain an acceptable level of subjective well-being. It seems reasonable to assume that living with locked-in syndrome would frustrate many of the critical interests a healthy person might have—achievement in one’s career, the completion of long-term projects, a close relationship with one’s family—which leads to the further assumption that such a life is not worth living. The fact that some locked-in patients report being happy suggests that the frustration of their prior critical interests has not significantly impacted their happiness. The same might be true for patients with covert awareness.

I have argued that there is evidence to suggest that patients with covert awareness could retain the capacity for critical interests in the present (i.e., to have a sense of what is meaningful or valuable in life, for them), which may be different from their past critical interests. This possibility gives us reason to question the authority of their past critical interests in decisionmaking on their behalf. A patient’s past critical interests may reflect how they wanted their lives to go in the past, but may not reflect how they want their lives to go in the present. Similarly, if we benefit a patient by considering the character of their life as a whole, we ought to defer to the critical interests they have in the present, rather than those they expressed in the past but no longer hold.

Where does this leave surrogate decisionmakers? As I discussed at the outset, the presence of covert awareness means that patients have some experience of the world, although we have limited knowledge of what this experience is like. Accordingly, it is unclear what is most consistent with their experiential interests, that is, whether continuing to provide life-sustaining treatment will result in a favorable balance of positive experiences (e.g., pleasure, enjoyment) over negative experiences (e.g., pain, emotional or mental suffering). But as we have seen, we should also be cautious about placing too much weight on the desires, preferences, and values that they expressed in the past. This may not be the best way to respect their autonomy, preserve their life-narrative, or help them to conclude their lives “the way they want.” At the same time, surrogates likely will not have any hard evidence for the patient’s critical interests in the present, if indeed they have undergone a revision.

Nevertheless, the reality is that treatment decisions will have to be made, and surrogates must make their decisions based on the evidence that is available to them. A patient’s past critical interests might be good evidence of their critical interests in the present, although this cannot be assumed to be the case. Some critical interests might be more likely to undergo revision than others. Consider again the testimony of patients with locked-in syndrome. In 2012, Tony Nicklinson petitioned the High Court of Justice of England and Wales for the right to die, after spending more than 6 years in a locked-in state he described as “dull, miserable, demeaning, undignified, and intolerable,” and “a living nightmare.”^58^ He died shortly after losing his case, having refused food for nearly a week.^59^ Another patient, Nick Chisholm, describes the “immense frustration” at completing the simplest of tasks, like having his teeth brushed, and the difficulty in communicating to others. He describes his existence as “incredibly lonely,” and that sometimes he wishes he had not survived his injury. Despite this, he is “glad to still be alive—most of the time anyway,” having come to accept his accident, and determined to make the most of his life.^60^ A third patient, Kevin Weller, says that he “felt unimaginable grief for the person I had lost—the old me,” yet he “does feel happy,” and “wishes to remain here as long as possible,” with “so much to look forward to.”^61^ The testimony of other patients emphasizes the importance of physical and emotional support from family, particularly in interpreting very subtle attempts at communication.^62^ Richard Marsh, who went on to recover completely after spending several months in a completely locked-in state, described “the loneliness of knowing there is no one there who understands how to communicate with you,” and feeling “very vulnerable” to carers, particularly those who were less skilled and handled him roughly.^63^

Each of the patients described has experienced their disability differently, and it seems reasonable to infer that the same would be true of patients with covert awareness. Accordingly, there should not be a presumption in favor of, or against, the provision of life-prolonging treatment. The locked-in patients described above also consistently refer to the simple experiences of day-to-day life as being the source of much of their frustration, as well as much of their enjoyment. It is their experiential interests which appear to have the largest impact on their well-being, whether they describe their lives as broadly negative, broadly positive, or as intolerable. This suggests that locked-in patients are less concerned with how their disability has impacted the overall narrative of their lives—broadly, with how it has affected their critical interests—and understandably much more focused on overcoming the immediate struggles they face, or enjoying the pleasures that are available to them. Again, although covertly aware patients might experience their disability differently than locked-in patients experience theirs, it is plausible that their experiential interests might have a similarly greater impact on their well-being than their critical interests.

If this is the case, surrogate decisionmakers should give greater consideration to promoting the present and future experiential interests of patients, rather than their critical interests. How might the patient experience a life of near-complete dependence on others, and how likely are they to be able to accept their limitations? For example, a patient’s ability to cope with severe disability has been found to be a combination of attributional style (perceived source of stress, locus of control, optimistic or pessimistic outlook), personality characteristics (e.g., risk tolerance, sense of self-efficacy, introversion or extroversion), and the implementation of coping strategies.^64^ ^,^^65^ Research has also found a strong relationship between family functioning and patient progress in postacute rehabilitation, suggesting the coping style of families can impact a patient’s functional recovery.^66^ The extent and quality of the care that can be provided to patients and the capacity of families to deal with the challenges of caregiving, should be an important consideration in treatment decisions, given its impact on patient well-being. Appropriate surrogate decisionmaking in this context requires making difficult judgments about patient quality of life. This will involve an accounting of the critical interests a patient may still endorse, and their experiential interests. Recently developed tools for assessing quality of life in patients with covert awareness may be useful in helping surrogate decisionmakers to focus on those aspects of well-being that are most relevant to patients as they exist in the present.^67^

## Conclusions

Behaviorally nonresponsive patients with covert awareness challenge our moral imagination like few other patient groups. Many assume that these patients are suffering, yet we have limited insight into their actual experience. As healthy people, we can imagine what we would want if we were in such a state, but we might feel differently once it becomes a reality. I have argued that when it comes to making decisions on behalf of patients with covert awareness, we have reason for skepticism about the authority of their past decisions, or previous critical interests. Appeal to these interests may be appropriate when making decision on behalf of a patient we know is no longer autonomous, and incapable of revising their values or desired life-narrative, such as in cases of advanced dementia. However, we cannot rule out the possibility that covertly aware patients have (or will in the future) revised their critical interests or life-narrative, and there is some positive evidence to suggest that they could. There is also reason to think that in these patients, it is their experiential interests which have a greater impact on well-being. Rather than attempting to reconstruct what a patient “would have wanted,” surrogate decisionmakers should reflect on how the patient is likely to experience their lives, now and in the future.

## Notes

1.

Cavinato M , Freo U , Ori C , Zorzi M , Tonin P , Piccione F , et al. Post-acute P300 predicts recovery of consciousness from traumatic vegetative state. Brain Injury 2009;23(12):973–80.1983149410.3109/02699050903373493

2.

Royal College of Physicians. Prolonged Disorders of Consciousness: National Clinical Guidelines. London, UK: RCP; 2013.10.7861/clinmedicine.14-1-4PMC587361824532734

3.

Schnakers C , Vanhaudenhuyse A , Giacino J , Ventura M , Boly M , Majerus S , et al. Diagnostic accuracy of the vegetative and minimally conscious state: Clinical consensus versus standardized neurobehavioral assessment. BMC Neurology 2009;9:35.1962213810.1186/1471-2377-9-35PMC2718857

4.

Owen AM , Coleman MR , Boly M , Davis MH , Laureys S , Pickard JD . Detecting awareness in the vegetative state. Science 2006;313(5792):1402.1695999810.1126/science.1130197

5.

Monti M , Vanhaudenhuyse A , Coleman MR , Boly M , Pickard JD , Tshibanda L , et al. Willful modulation of brain activity in disorders of consciousness. New England Journal of Medicine 2010;362:579–89.2013025010.1056/NEJMoa0905370

6.

Cruse D , Chennu S , Chatelle C , Bekinschtein TA , Fernandez-Espejo D , Pickard JD , et al. Bedside detection of awareness in the vegetative state: A cohort study. The Lancet 2011;378(9809):2088–94.10.1016/S0140-6736(11)61224-522078855

7.

Stender J , Gosseries O , Bruno MA , Charland-Verville V , Vanhaudenhuyse A , Demertzi A , et al. Diagnostic precision of PET imaging and functional MRI in disorders of consciousness: A clinical validation study. The Lancet 2014;384(9942):514–22.10.1016/S0140-6736(14)60042-824746174

8.

Kondziella D , Friberg CK , Frokjaer VG , Fabricius M , Moller K . Preserved consciousness in vegetative and minimally conscious states: Systematic review and meta-analysis. Journal of Neurology, Neurosurgery, and Psychiatry 2016;87(5):485–92.10.1136/jnnp-2015-31095826139551

9.

Schiff ND . Cognitive motor dissociation following severe brain injuries. JAMA Neurology 2015;72(12):1413–5.2650234810.1001/jamaneurol.2015.2899

10.

Naci L , Cusack R , Anello M , Owen AM . A common neural code for similar conscious experiences in different individuals. Proceedings of the National Academy of Science USA 2014;111(39):14277–82.10.1073/pnas.1407007111PMC419178225225384

11.

Graham M , Naci L , Owen AM , Weijer C . Covert narrative capacity: Mental life in patients thought to lack consciousness. Annals of Clinical and Translational Neurology 2017;4(1):61–70.2807831610.1002/acn3.376PMC5221458

12.

Fernandez-Espejo D , Owen AM . Detecting awareness after severe brain injury. Nature Reviews Neuroscience 2013;14(11):801–9.2408881010.1038/nrn3608

13. See note 2, Royal College of Physicians 2013.

14.

Giacino JT , Katz DI , Schiff ND , Whyte J , Ashman EJ , Ashwal S , et al. Practice guideline update recommendations summary: Disorders of consciousness. Neurology 2018;91(10):450–60.3008961810.1212/WNL.0000000000005926PMC6139814

15.

Kahane G , Savulescu J . Brain damage and the moral significance of consciousness. Journal of Medicine and Philosophy 2009;34(1):6–26.1919369410.1093/jmp/jhn038PMC3242047

16.

Hawkins J. What is good for them? Best interests and severe disorders of consciousness In: Sinott-Armstrong W , ed. Finding Consciousness: The Neuroscience, Ethics, and Law of Severe Brain Damage. Oxford: Oxford University Press; 2016, at 180–200.

17.

Berger JT , DeRenzo EG , Schwartz J . Surrogate decision making: Reconciling ethical theory and clinical practice. Annals of Internal Medicine 2008;149(1):48–53.1859163710.7326/0003-4819-149-1-200807010-00010

18.

Torke AM , Alexander GC , Lantos J . Substituted judgement: The limitations of autonomy in surrogate decision making. Journal of General Internal Medicine 2008;23(9):1514–7.1861820110.1007/s11606-008-0688-8PMC2518005

19.

Hirschmann KB , Kapo JM , Karlawish JHT . Why doesn’t a family member of a person with advanced dementia use a substituted judgement when making a decision for that person? The American Journal of Geriatric Psychiatry 2006;14(8):659–67.1686137010.1097/01.JGP.0000203179.94036.69

20. See note 17, Berger et al. 2008.

21.

Long B , Clark L , Cook P . Surrogate decision making for patients with severe traumatic brain injury. Journal of Trauma Nursing 2011;18(4):204–12.2215752810.1097/JTN.0b013e31823a453a

22.

Devnani R , Slaven JE , Bosslet GT , Montz K , Inger L , Burke ES , et al. How surrogates decide: A secondary data analysis of decision making principles used by the surrogates of hospitalized older adults. Journal of General Internal Medicine 2017;32(12):1285–93.2884048510.1007/s11606-017-4158-zPMC5698224

23.

Dworkin R. Life’s Dominion: An Argument about Abortion and Euthanasia. London, UK: Harper Collins; 1993.

24. See note 23, Dworkin 1993, at 226.

25.

Rao JK , Anderson LA , Lin FC , Laux JP . Completion of advance directives among US consumers. American Journal of Preventive Medicine 2014;46(1):65–70.2435567310.1016/j.amepre.2013.09.008PMC4540332

26.

Yadav KN , Gabler NB , Cooney E , Kent S , Kim J , Herbst N , et al. Approximately one in three US adults completes any type of advance directive for end-of-life care. Health Affairs 2017;36(7):1244–51.2867981110.1377/hlthaff.2017.0175

27.

Fischer GS , Tulsky JA , Rose MR , Siminoff LA , Arnold RM . Patient knowledge and physician predictions of treatment preferences after discussion of advance directives. Journal of General Internal Medicine 1998;13(7):447–54.968671010.1046/j.1525-1497.1998.00133.xPMC1496983

28.

Thorevska N , Tilluckdharry L , Tickoo S , Havasi A , Amoateng-Adjepong Y , Manthous CA . Patients understanding of advance directives and cardiopulmonary resuscitation. Journal of Critical Care 2005;20(1):26–34.1601551410.1016/j.jcrc.2004.11.002

29.

Wilson TD , Gilbert DT . Affective forecasting: Knowing what to want. Current Directions in Psychological Science 2005;14(3):131–4.

30.

Loewenstein G , Ubel PA . Hedonic adaptation and the role of decision and experience utility in public policy. Journal of Public Economics 2008;92:1795–810.

31.

Lambert HC , McColl MA , Gilbert J , Wong J , Murray G , Shortt SED . Factors affecting long-term care residents’ decision making processes as they formulate advance directives. The Gerontologist 2005;45(5):626–33.1619939710.1093/geront/45.5.626

32.

Fagerlin A , Ditto PH , Hawkins NA , Schneider CE , Smucker WD . The use of advance directives in end-of-life decision making: Problems and possibilities. American Behavioural Scientist 2002;46(2):268–83.

33.

Fried TR , Byers AL , Gallo WT , Van Ness PH , Towle VR , O’Leary JR , et al. Prospective study of health status preference changes over time in older adults. Archives of Internal Medicine 2006;166(8):890–5.1663621510.1001/archinte.166.8.890PMC1978221

34.

Auriemma CL , Nguyen CA , Bronheim R , Kent S , Nadiger S , Pardo D , et al. Stability of end-of-life preferences: A systematic review of the evidence. JAMA Internal Medicine 2014;174(7):1085–92.2486156010.1001/jamainternmed.2014.1183PMC8243894

35. See note 17, Berger et al. 2008.

36.

Breslin J. The status quo bias and decision to withdraw life-sustaining treatment. Canadian Medical Association Journal 2018;190(9):E265–7.10.1503/cmaj.171005PMC583787630986193

37.

Phillips J , Wendler D . Clarifying substituted judgement: The endorsed life approach. Journal of Medical Ethics 2015;41:723–30.2536002910.1136/medethics-2013-101852

38.

Shalowitz DI , Garrett-Mayer E , Wendler D . The accuracy of surrogate decision-makers: A systematic review. JAMA Internal Medicine 2006;166(5):493–7.10.1001/archinte.166.5.49316534034

39. See note 23, Dworkin 1993, at 224.

40. See note 23, Dworkin 1993, at 225.

41. Rebecca Dresser has made a similar argument that an incompetent patient should not be treated according to her former wishes, but according to her interests in the present. Although I broadly agree with Dresser’s conclusion, my argument differs from hers in a number of ways. Dresser accepts that most patients lacking decisionmaking capacity will have abandoned the kinds of critical interests that Dworkin emphasizes once they become incompetent; what is central to their well-being are the kinds of “simple pleasures” that they can continue to appreciate. Thus, the conflict is between a patient’s present broadly experiential interests, and their past critical interests; she argues we should privilege their present experience over their past critical interests. But this is susceptible to the counter-argument that even if incompetent patients in the present do not care about their past critical interests, satisfying these interests might still be in their best interests when considering their lives as a whole. Conversely, I argue that patients with covert awareness may continue to have critical interests in the present, and this is why the prior expression of their critical interests may lack authority. However, because we are uncertain of what these present critical interests are, surrogate decision-makers must also rely on their experiential interests.

42. See note 37, Phillips et al. 2015.

43.

Brudney D , Lantos J . Agency and authenticity: Which value grounds patient choice? Theoretical Medicine and Bioethics 2011;32(4):217–27.2150950610.1007/s11017-011-9180-2

44. See note 43, Brudney et al. 2011, at 221.

45. See note 12, Fernandez-Espejo et al. 2013, at 802.

46. See note 12, Fernandez-Espejo et al. 2013.

47. See note 10, Naci et al. 2014.

48. See note 11, Graham et al. 2017.

49.

Chow HM , Kaup B , Raabe M , Greenlee MW . Evidence of pronto-temporal interactions for strategic inference processes during language comprehension. NeuroImage 2007;40:940–54.1820191110.1016/j.neuroimage.2007.11.044

50.

Lehne M , Engel P , Rohrmeier M , Menninghaus W , Jacobs AM , Koelsch S . Reading a suspenseful literary text activates brain areas related to social cognition and predictive inference. PLoS One 2015;10(5):e0124550.2594630610.1371/journal.pone.0124550PMC4422438

51.

Schwartz CE , Sprangers MA . Methodological approaches for assessing response shift in longitudinal health-related quality-of-life research. Social Science and Medicine 199(11):1531–48.10.1016/s0277-9536(99)00047-710400255

52.

McClimans L , Bickenbach J , Westerman M , Carlson L , Wasserman D , Scwartz C . Philosophical perspectives on response shift. Quality of Life Research 2013;22(7):1871–78.2310408910.1007/s11136-012-0300-x

53.

Blome C , Augustin M . Measuring change in quality of life: Bias in prospective and retrospective evaluation. Value Health 2015;18(1):110–5.2559524110.1016/j.jval.2014.10.007

54.

Laureys S , Pellas F , Van Eeckhout P , Ghorbel S , Schnakers C , Perrin F , et al. The locked-in syndrome: What is it like to be conscious but paralyzed and voiceless? Progress in Brain Research 2005;150:495–511.1618604410.1016/S0079-6123(05)50034-7

55.

Lulé D , Zickler C , Häcker S , Bruno MA , Demertzi A , Pellas F , et al. Life can be worth living in locked-in syndrome. Progress in Brain Research 2009;177:339–51.1981891210.1016/S0079-6123(09)17723-3

56.

Bruno MA , Bernheim JL , Ledoux D , Pellas F , Demertzi A , Laureys S . A survey on self-assessed well-being in a cohort of chronically locked-in patients: Happy majority, miserable minority. BMJ Open 2011;1:e000039.10.1136/bmjopen-2010-000039PMC319140122021735

57. See note 56, Bruno et al. 2011.

58.

Topping A , Jones S . Locked-in syndrome sufferer wins high court hearing for his right to die. *The Guardian* March 12, 2012; available at https://www.theguardian.com/society/2012/mar/12/locked-in-syndrome-sufferer-court-hearing (last accessed 5 Feb 2020).

59.

Gallagher J . Right-to-die man Tony Nicklinson dead after refusing food. *BBC News* August 22, 2012; available at https://www.bbc.co.uk/news/uk-england-19341722 (last accessed 5 Feb 2020).

60.

Chisholm N , Gillett G . The patient’s journey: Living with locked-in syndrome. BMJ 2005;331:94–7.1600288410.1136/bmj.331.7508.94PMC558618

61.

Weller K , Clark J . ‘I do feel happy’—Living with locked in syndrome. *The Guardian* March 29, 2011; available at https://www.theguardian.com/lifeandstyle/2011/mar/29/happy-with-locked-in-syndrome (last accessed 6 Feb 2020).

62.

Rohrer F. How do people cope with locked-in syndrome? *BBC News* November 25, 2009; available at http://news.bbc.co.uk/1/hi/magazine/8378262.stm (last accessed 6 Feb 2020).

63.

Hill A. Locked-in syndrome: Rare survivor Richard Marsh recounts his ordeal. *The Guardian* August 7, 2012; available at https://www.theguardian.com/world/2012/aug/07/locked-in-syndrome-richard-marsh (last accessed 6 Feb 2020).

64.

Gregorio GW , Ponds RWHM , Smeets SMJ , Jonker F , Pouwels CGJG , Verhey FR , et al. Associations between executive functioning, coping, and psychosocial function after acquired brain injury. British Journal of Clinical Psychology 2015;54(3):291–306.2558639310.1111/bjc.12074

65.

Boosman H , Winkens I , van Heugten CM , Rasquin SM , Heijnen VA , Visser-Meily JM . Predictors of health-related quality of life and participation after brain injury rehabilitation: The role of neuropsychological factors. Neuropsychological Rehabilitation 2017;27(4):581–98.2660979810.1080/09602011.2015.1113996

66.

Haller CS . Twelve month prospective cohort study of patients with severe traumatic brain injury and their relatives: Coping, satisfaction with life and neurological functioning. Brain Injury 2017;13–14:1903–9.10.1080/02699052.2017.134629528829653

67.

Tung J , Speechley KN , Gofton T , Gonzalez-Lara L , Graham M , Naci L , et al. Towards the assessment of quality of life in patients with disorders of consciousness. Quality of Life Research 2019. doi:10.1007/s11136-019-02390-8. [Epub ahead of print].31838655

